# A Meta-Analysis of MicroRNA Expression in Liver Cancer

**DOI:** 10.1371/journal.pone.0114533

**Published:** 2014-12-09

**Authors:** Jingcheng Yang, Shuai Han, Wenwen Huang, Ting Chen, Yang Liu, Shangling Pan, Shikang Li

**Affiliations:** 1 First Affiliated Hospital of Guangxi Medical University, Nanning, Guangxi Zhuang Autonomous Region, China; 2 Department of Management Information System, College of Computer and Information Engineering, Guangxi Teachers Education University, Nanning, Guangxi Zhuang Autonomous Region, China; 3 Department of Pathophysiology, Guangxi Medical University, Nanning, Guangxi Zhuang Autonomous Region, China; CRCL-INSERM, France

## Abstract

MicroRNA (miRNA) played an important role in the progression of liver cancer and its diagnostic and prognostic values have been frequently studied. However, different microarray techniques and small sample size led to inconsistent findings in previous studies. We performed a comprehensive meta-analysis of a total of 357 tumor and 283 noncancerous samples from 12 published miRNA expression studies using robust rank aggregation method. As a result, we identified a statistically significant meta-signature of five upregulated (miR-221, miR-222, miR-93, miR-21 and miR-224) and four downregulated (miR-130a, miR-195, miR-199a and miR-375) miRNAs. We then conducted miRNA target prediction and pathway enrichment analysis to find what biological process these miRNAs might affect. We found that most of the pathways were frequently associated with cell signaling and cancer pathogenesis. Thus these miRNAs may involve in the onset and progression of liver cancer and serve as potential diagnostic and therapeutic targets of this malignancy.

## Introduction

Liver cancer in men is the second most frequent cause of cancer death and is the sixth leading cause of cancer death in women, which causes approximately 700 thousand deaths per year with about 750 thousand new cases diagnosed worldwide. [1] Low survival is attributed to late diagnosis, resistance to chemotherapy, tumor recurrence, and metastasis, hence stressing the need for novel diagnostics and therapeutics. Numerous gene expression studies have shown that a general aberrant activation of signaling pathways was attributed to the oncogenicity, however, one signature or a single prominent characteristic pathway could not be defined in liver cancer. [2]

MicroRNAs (miRNAs), small non-coding RNAs of 18–25 nucleotides in length, which were seen to control gene expression in virtually all cancer cells, were abundantly investigated relating to the occurrence, progress, classification, diagnosis and treatment of tumors recently. The involvement of miRNAs in cancer pathogenesis is well established, as they can behave as oncogenes or tumor suppressor genes depending on the cellular function of their targets. [3] Understanding the biology of miRNA and its contribution to cancer development may promise early diagnosis and effective control of malignant tumors.

Recent findings from integrative and mechanism-based profiling studies have provided important information about the roles of miRNAs in normal cells and disease condition. These studies could improve our understanding of the molecular mechanisms of chronic liver diseases and liver cancer. Numerous studies linking to the deregulation of miRNA expression to liver cancer have been reported with multifarious methods. [4] However, due to the application of different technological platforms and small sample size, the miRNA expression profiling efforts have led to inconsistent results between the studies.

To overcome the limitations in current researches, we performed a meta-analysis applying the robust rank aggregation method, [5] followed by pathway analysis, to identify miRNA deregulation in liver cancer and the pathways that key miRNAs may impact. The leave-one-out cross-validation method was used to validate the results. Identification of miRNA meta-signature and invovled pathways would provide potential targets for further experimental studies of liver cancer development.

## Material and Method

### Study selection and data extraction

A systematic literature search was performed for the identification of liver cancer miRNA expression profiling studies using a two-level search strategy. First, we undertook a web-based search in Gene Expression Omnibus (GEO, www.ncbi.nlm.-nih.gov/geo/) using search term (("Neoplasms"[Mesh] AND "Liver"[Mesh]) AND "MicroRNAs"[Mesh]) AND "Humans"[Mesh]. To perform a comprehensive retrieval, searching in ArrayExpress (www.ebi.ac.uk/arrayexpress), the Pubmed and the Embase database were also performed. Second, the reference lists of all relevant and existing studies were reviewed through a manual search for further identification of potential relevant studies.

Abstracts were screened carefully and full texts of relevant potential abstracts were evaluated. Studies with original experimental design that analyzed the miRNA expression profiling in human between liver cancer tissues and non-tumorous liver tissues were included. Meanwhile, studies were not eligible for meta-analysis if they met the following selection criteria: 1) using only cell lines, 2) preselected candidate genes research, 3) profiling different clinical or histologic subtypes without including non-tumorous tissues.

Lists of statistically significant expressed miRNAs were extracted from publications. Authors were contacted when the lists could not be obtained. All miRNA names were standardized through miRBase version 20. MiRNAs that cannot be related to either -3p or -5p in miRBase were designated with hsa-miR-*, such as hsa-miR-210. Those identified as dead entry in miRBase were remained their names mentioned in the literatures.

### Statistical analysis

The lists of miRNAs were extracted based on statistical test p-values (<0.05 was considered significant). Then, miRNAs in every list were prioritized by fold changes or other values that could indicate the degree of deregulation. To ensure that extracted miRNAs can be ranked in a more reliable way, we used robust rank aggregation method. [5] The method is based on the comparison of actual data with a null model that assumes random order of input lists. A P-value assigned to each element in the aggregated list described how much better it was ranked than expected. In case of false positive results, Bonferroni correction was performed. Meanwhile, to assess the stability of acquired p-values, leave one out cross-validation was applied on the robust rank aggregation algorithm. An averaged p-value was obtained from random gene lists after repeating the analyses 10,000 times.

### Clustering analysis

To investigate the correlations among the miRNA expression profiles of individual studies, we performed hierarchical clustering using the deregulated miRNAs. Two-dimensional average-linkage hierarchical clustering of a Spearman rank correlation similarity matrix constructed from separate analyses for upregulated and downregulated gene lists was performed.

### Integrative identification of miRNA targets

The meta-signature miRNAs were selected for target prediction by using TargetScan database version 6.2 [6], PicTar (predictions from miRWalk database) [7] and DIANA-microT-CDS v5.0 (using miTG score threshold 0.7) [8]. TarBase v6.0database [9] and CLIP-Seq database starBase [10] were used to acquire validated targets. To improve the accuracy of the target prediction, consensus targets were extracted for the overlapping targets predicted by at least two algorithms plus validated targets from TarBase and starBase.

### Enrichment analysis

To identify the pathways of predicted miRNA targets, Kyoto Encyclopedia of Genes and Genomes (KEGG), Panther pathways and Gene Ontology terms were carried out with GeneCodis web tool (http://genecodis.dacya.ucm.es/). [11]

## Results

### Study selection and data extraction

**Figure 1:**
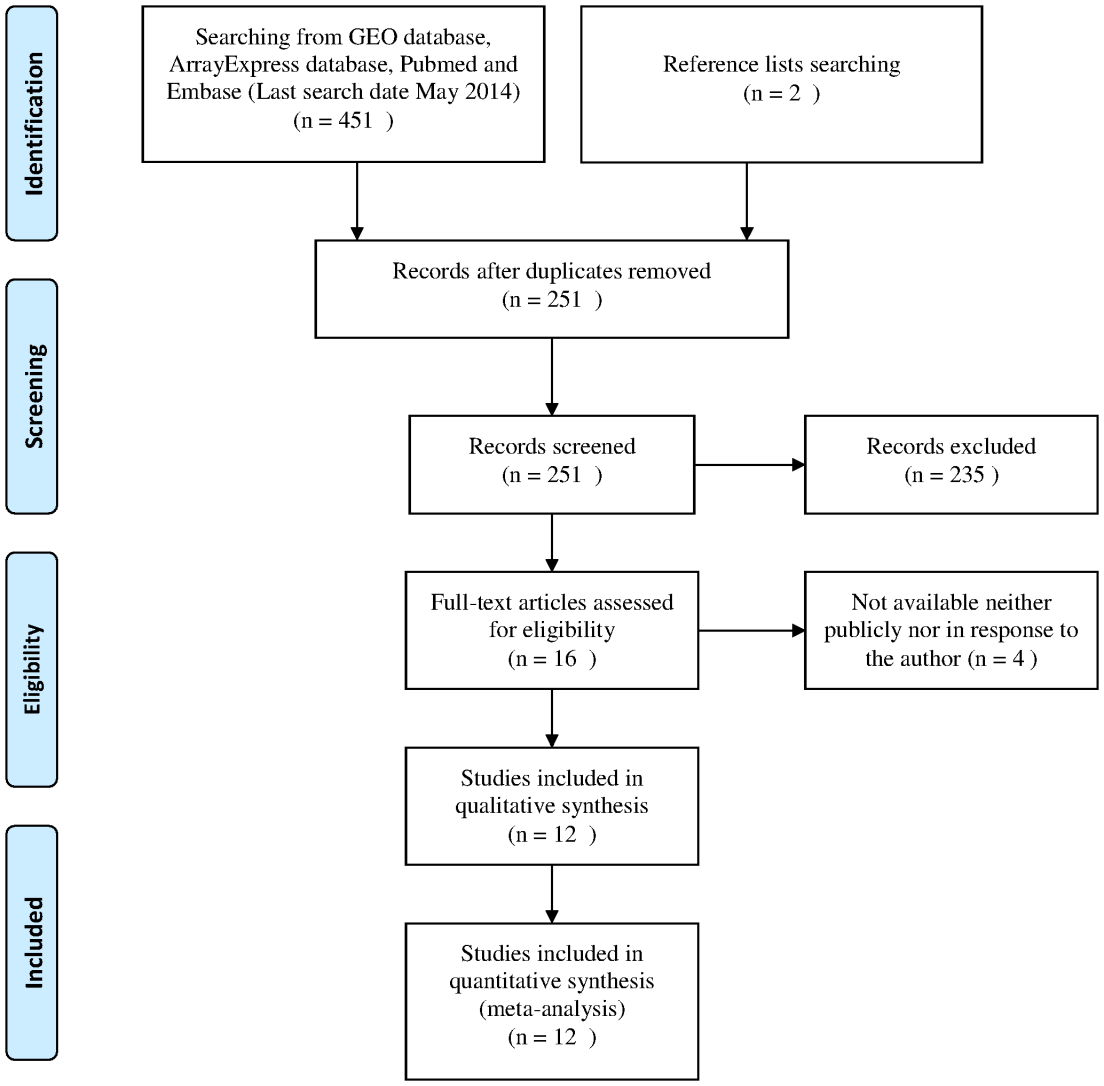
Searching strategy.

Database searches initially yielded a total of 251 publications and 16 studies met the inclusion criteria. (Fig. 1) Four researches [12]–[15] were excluded in the final analysis because the lists of ranked miRNA were not neither publicly nor personally available from the corresponding authors. Most of the studies were published between 2009 and 2013. Two-thirds of the researches came from Asian region and the remainings were come from America and Europe. Moreover, included studies used various microarray platforms and the average number of miRNA probes was 626 (ranging from 121 to 1205). A total of 357 tumor and 283 noncancerous samples were included. The majority of the studies focused on hepatocellular carcinoma (HCC) compared with adjacent nontumorous tissues or normal liver tissues except Cario 2010 and Selaru 2009, which respectively focused on hepatoblastoma (HB) and cholangiocarcinoma (CCA). The main characteristics of these studies were listed in Table 1.

**Table 1:** Characteristics of included studies.

Study	Platform	Probes of miRNAs	Cancer type	Samples		Type of samples	Region
				Tumor	Control		
Cairo 2010 [41]	OSU-CCC miRNA microarray v2.0	429	HB	49	7	49 TT +7 NL	France,Europe
Chung 2010 [42]	Rosetta Genomics Corp RNA microarray	308	HCC	25	25	25 pairs	Korea,Asia
Diaz 2013 [17]	Affymetrix GeneChip miRNA2.0 arrays	1205	(HCV+) HCC	9	19	9 TT +19 NL	USA,North America
Elyakim 2010 [43]	custom microarrays	474	HCC	30	30	30 pairs	Israel,Asia
Koh 2013 [44]	Genomic Tree	939	HCC	4	4	4 pairs	Korea,Asia
Li 2008 [45]	CapitalBio Mammalian miRNA Array Services v1	121	HCC	78	88	78 pairs +10 NL	China,Asia
Meng 2007 [46]	Ambion mirVana Bioarray v2	328	HCC	3	3	3 pairs	USA,North America
Sato 2011 [47]	3D-Gene Human miRNA Oligo chip v12–1.00	934	HCC	73	73	73 pairs +4NL	Japan,Asia
Selaru 2009 [48]	Agilent Human miRNA Array	470	CCA	5	5	5 TT +5 NBD	USA,North America
Shih 2012 [49]	Illumina Human v2 MicroRNA expression beadchip	1145	HCC	68	21	68 TT +21 ANT	Taiwan,China,Asia
Su 2009 [50]	CapitalBio human/mouse/rat non-coding RNA microarray	308	HCC	5	3	3 pairs +2 ANT	China,Asia
Yang 2010 [51]	Exiqon miRCURY LNA array v11.0	856	(HBV+) HCC	8	5	8 TT +5 NL	China,Asia

Note: HCC = hepatocellular carcinoma, HB = hepatoblastoma, CCA =  cholangiocarcinoma, TT = tumor tissues, ANT = adjacent nontumorous tissues, NL = normal liver tissues, NBD = normal bile duct specimen, HBV =  hepatitis B virus, HCV =  hepatitis C virus, N.M. = not mentioned.

In total, 136 miRNAs were reported as significantly upregulated and 138 as significantly downregulated in included studies. The number of significantly deregulated miRNAs varied greatly across studies (ranging from 14 to 91).

### Cluster analysis

To assess the degree of concordance between miRNA lists and possible correlation according to the subgroups of tumor histology, region, and sample size, hierarchical clustering analysis was performed (S1 Figure). The clustering of these lists showed that the results of Shih 2012, Sato 2011 and Li 2008 using the HCC tissues were more similar to each other than any other studies. The most similar results were Li 2008-1 and Li 2008-2, conducted by the same workgroup using the same platform though the sample size differs greatly. It is not clear whether there was some overlap of the samples between the two studies, but if this is the case, it may be one explanation for the high similarity of the results. No more obvious similarities were seen between other subgroups.

### MiRNA meta-signature

We identified a statistically significant meta-signature of five upregulated miRNAs and four downregulated miRNAs in liver cancer samples compared to noncancerous liver tissue according to the permutation p-value. (Table 2) Only two upregulated but not downregulated miRNAs reached statistical significance after Bonferroni correction. The number of meta-signature miRNAs studies reported varied greatly but at least three deregulated miRNAs were reported by each study, with an exception of Cairo 2010 and Selaru 2009, which just separately reported two upregulated miRNAs. The most significantly deregulated miRNAs, miR-221, miR-222, are respectively reported by nine and ten datasets. Furthermore, the permutation p-values of another three upregulated miRNAs, miR-93, miR-21 and miR-224, and four downregulated miRNAs, miR-130a, miR-195, miR-199a and miR 375 are <0.05, but do not reach the corrected significance.

**Table 2:** Meta-signature miRNAs in liver cancer.

MiRNA	Robust rank aggregation p-value	Corrected p-vlaue	Permutation p-value	Studies	Chromosome
Upregulated					
hsa-miR-221-3p	2.26E-06	2.72E-03	1.84E-06	9	Xp11.3
hsa-miR-222-3p	2.60E-06	3.13E-03	2.33E-06	10	Xp11.3
hsa-miR-93-5p	0.000769994	9.28E-01	0.00527528	7	7q22.1
hsa-miR-21-5p	0.002230281	2.69E+00	0.004349022	6	17q23.1
hsa-miR-224-5p	0.012059959	1.45E+01	0.020557142	6	Xq28
Downregulated					
hsa-miR-130a-3p	0.00200451	2.42E+00	0.009886451	7	11q12.1
hsa-miR-195-5p	0.005465174	6.59E+00	0.022212131	7	17p13.1
hsa-miR-199a-5p	0.005851168	7.05E+00	0.003374759	7	19p13.2
hsa-miR-375	0.01036539	1.25E+01	0.042655185	3	2q35

Meta-signature miRNA genes are scattered on different chromosomal locations with an exception of miR-221 and miR-222 genes, which are both located on Xp11.3. MiR-224 genes are located in the same chromosomal region, Xq28, which is a cytogenetic region that contains a large portion of the cancer/testis antigen gene family. [16]

### Target prediction and enrichment analysis

**Figure 2:**
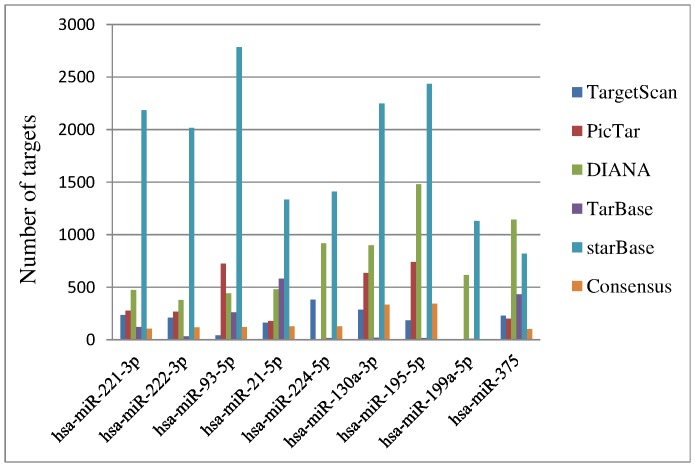
Target counts of meta-signature miRNAs.

Consensus targets for miRNAs reaching robust rank aggregation significance were extracted for the overlapping targets predicted by at least two algorithms plus experimentally validated targets from two databases. The summery of target counts is presented in Fig. 2. MiR-130a and miR-195 have more targets than other miRNAs, whereas miR-199a has no targets because it was predicted by only one algorithm.

**Figure 3:**
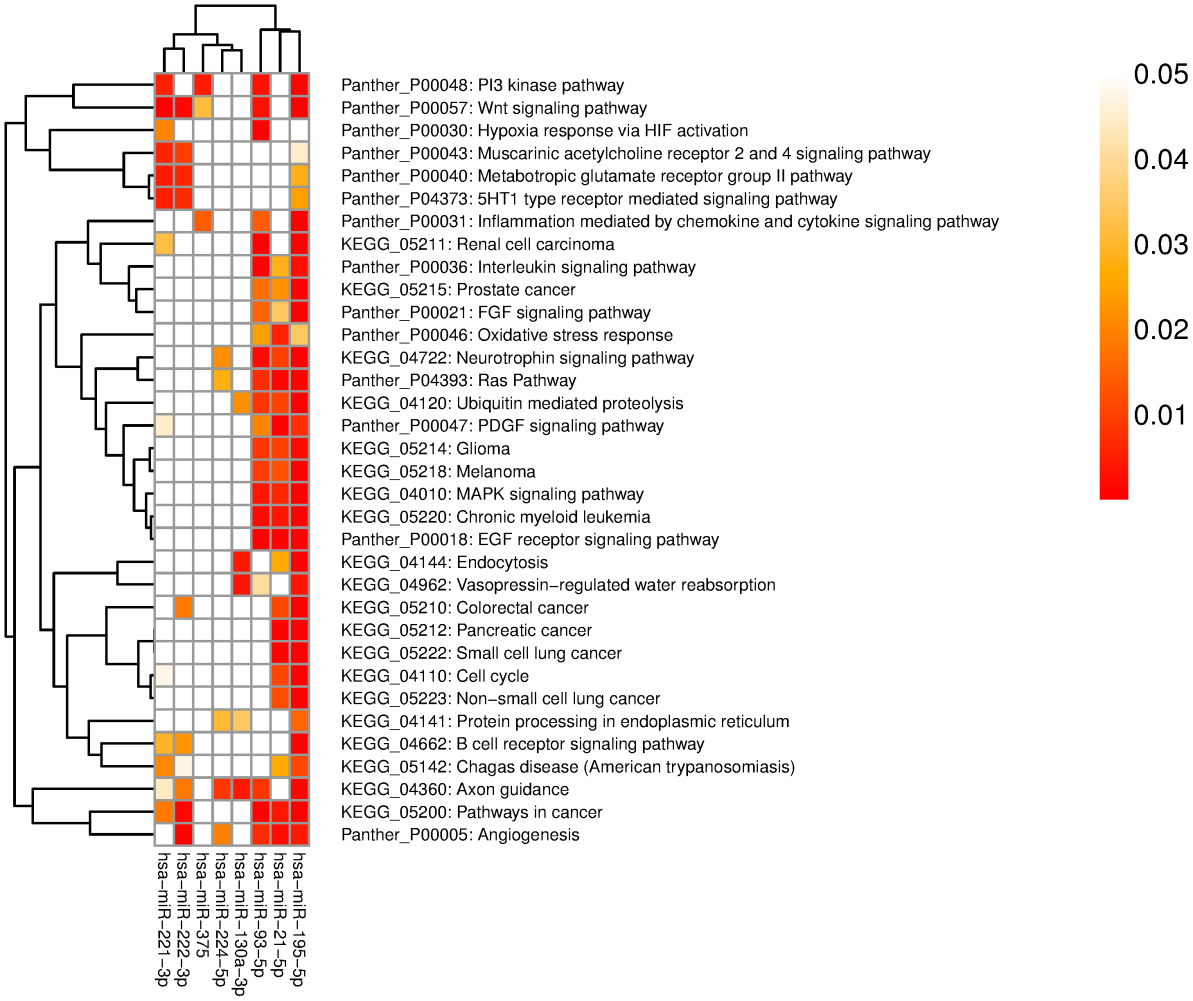
Pathway enrichment of meta-signature miRNA targets. The intensity of color represents the FDR-corrected p-value. Only those pathways, which were significant for more than four miRNAs are shown (full data are available as S2 Figure).

Enrichment analyses were performed using predicted target genes with GeneCodis web tool. Several pathways enriched by KEGG and Panther pathways were relatively significant and most of them were frequently associated with cell signaling (e.g. neurotrophin, Wnt, FGF, and p53 signaling pathway) and cancer. (Table 3, Fig. 3, S2 Figure)

**Table 3:** GO processes and pathways most strongly enriched by meta-signature miRNA targets.

Pathway enrichment analysis	FDR	Targets
GO process		
GO:0006355: regulation of transcription, DNA-dependent (BP)	3.34324E-13	39
GO:0007165: signal transduction (BP)	3.77687E-13	42
GO:0045944: positive regulation of transcription from RNA polymerase II promoter (BP)	1.84064E-11	22
GO:0051301: cell division (BP)	1.13136E-10	19
GO:0044419: interspecies interaction between organisms (BP)	1.67014E-10	20
GO:0007049: cell cycle (BP)	3.92822E-09	21
GO:0007399: nervous system development (BP)	5.35901E-09	12
GO:0007156: homophilic cell adhesion (BP)	8.576E-09	8
GO:0030154: cell differentiation (BP)	1.00415E-08	22
GO:0008285: negative regulation of cell proliferation (BP)	1.15719E-08	18
KEGG pathway		
Kegg:05200: Pathways in cancer	1.85246E-11	21
Kegg:04722: Neurotrophin signaling pathway	4.02366E-10	13
Kegg:04310: Wnt signaling pathway	3.9198E-09	13
Kegg:04510: Focal adhesion	1.10106E-07	13
Kegg:04115: p53 signaling pathway	3.36014E-07	8
Kegg:04110: Cell cycle	4.83318E-07	10
Kegg:05212: Pancreatic cancer	5.06487E-07	8
Kegg:05220: Chronic myeloid leukemia	7.02671E-07	8
Kegg:04120: Ubiquitin mediated proteolysis	1.1415E-06	10
Kegg:05221: Acute myeloid leukemia	1.61649E-06	7
Panther pathway		
Panther:P00057: Wnt signaling pathway	5.18994E-10	18
Panther:P00021: FGF signaling pathway	1.24487E-08	11
Panther:P00053: T cell activation	8.93741E-08	9
Panther:P00010: B cell activation	2.05044E-06	7
Panther:P00012: Cadherin signaling pathway	4.86842E-06	6
Panther:P00031: Inflammation mediated by chemokine and cytokine signaling pathway	5.58894E-06	11
Panther:P00047: PDGF signaling pathway	9.10927E-06	6
Panther:P00013: Cell cycle	1.13184E-05	3
Panther:P00012: Cadherin signaling pathway	1.16394E-05	9
Panther:P00005: Angiogenesis	0.000016605	6

## Discussion

Using robust rank aggregation method, 14 prioritized miRNA lists from the 12 published studies were analyzed and finally identified five upregulated and four downregulated miRNAs. But after Bonferroni correction only two upregulated miRNAs reached the statistical difference.

The deregulated miRNAs identified by different research centers did not allow a consistent conclusion. Differences among microarray techniques, stage of the tumor, histologic appearance and etiological factors attributed to the heterogeneity. [17] There have been attempts to divide the datasets into subgroups on the basis of platform and tumor-type subtype, but none of two studies have used same platform and the majority of tumor samples were from HCC tissues. However, the cluster analysis demonstrated that two datasets conducted by one work group used an identical platform made high similarity and anther meta-analysis used robust rank aggregation method also led to the same conclusion as us. [18]

In this study, we used the Bonferroni method to control the false positive rate and made the results more reliable. Ultimately, miR-221 and miR-222 were only two statistically significant meta-signature miRNAs. Furthermore, the permutation p-values of another three upregulated (miR-93, miR-21 and miR-224) and four downregulated miRNAs (miR-130a, miR-195, miR-199a and miR-375) were <0.05, but their corrected p-values were not significant. The following limitations may explain these finding: 1) there were not sufficient datasets for integration, 2) the sample sizes of the datasets were relatively small, 3) different methodology researchers used made more discrepant.

Although no strong significance was seen in all meta-signature miRNAs, experimental studies in recent years demonstrated that they were highly associated with liver cancer. MiR-221 was the most frequently studied among the meta-signature miRNAs. Fornari et al. [19] have proved that miR-221 functioned as an oncogene in hepatocarcinogenesis by targeting CDKN1B/p27 and CDKN1C/p57 in 2008. Meanwhile, results of Gramantieri et al. [20] have indicated that miR-221 inhibited apoptosis by targeting Bmf and its overexpression was associated with a more aggressive phenotype in 2009. Together, these findings implicated that miR-221 might be a potential target for nonconventional treatment against HCC. In addition, Park et al. [21] found that miR-221 silencing blocked hepatocellular carcinoma and promoted survival in a valid orthotopic mouse model of HCC and Callegari et al. [22] and Pineau et al. [23] have respectively confirmed the oncogenic role of miR-221 in the mice model. Moreover, Li et al. [24] demonstrated that serum miR-221 might provide predictive significance for prognosis of HCC patients and He et al. [25] have proved that miR-221 silencing inhibited liver cancer malignant properties in vitro and in vivo. Together, miR-221 has been deeply studied on its impact on liver cancer, and it might be a potential candidate target for liver cancer diagnosis and therapy.

Besides miR-221, some other miRNAs have been experimentally proved to associate with the occurrence, development, and metastasis. Three upregulated meta-signature miRNAs, miR-222, miR-21 and miR-224, might exacerbate HCC through AKT signaling pathways. [26]-[28] MiR-222 overexpression is common in HCC and could confer metastatic potentials in HCC cells possibly by enhancing AKT signaling. [26] MiR-21 suppresses PTEN and hSulf-1 expression and promotes HCC progression through AKT/ERK pathways, and as for miR-224, it possibly actives the AKT signaling pathways by targeting PPP2R1B. [27], [28] A recent study showed that miR-21 and miR-222 expressions were differentially modulated by Hepatitis B Virus X protein in malignant hepatocytes. [29] In addition, miR-224 might play significant role in migration and invasion of HCC cell. [30]-[32] Interestingly, two downregulated meta-signature miRNAs, miR-195 and miR-375, might plays important inhibitory roles in HCC progression. [33]–[38] For example, miR-195 might inhibit HCC progression by targeting LATS2 and the NF-κB signaling pathway [33], [34] and suppress angiogenesis and metastasis of hepatocellular carcinoma by inhibiting the expression of VEGF, VAV2, and CDC42. [35] Xu et al. have proved that miR-195 played an important role in cell cycle control and in the molecular etiology of HCC. [36] Moreover, miR-195 may target PCMT1 in hepatocellular carcinoma that increases tumor life span [39] and negatively regulate protein levels of steroid receptor coactivator-3 through targeting its 3'-untranslated region in HCC cells [40]. Another downregulated miRNA, miR-375, also has been proved to suppress liver cancer cell growth in vitro or in vivo. [37], [38]

Although we have demonstrated that miRNAs may involve in promoting or inhibiting liver cancer progression by targeting some genes in the key pathways of cancer regulation, there is still a long way to go in interpreting the impact of miRNA on liver cancer. Future work should keep continuous focus on the mechanism through which miRNAs regulate occurrence, progression and metastasis of liver cancer. In addition, studies with large sample size and the same platform are needed.

In conclusion, we suggest that the meta-signature miRNAs are key regulatory drivers of the oncogenic process, which may be good potential targets for the diagnosis and therapy of liver cancer.

## Supporting Information

S1 Figure **Cluster analysis of miRNA list.** Possible correlation was shown according to the subgroups of tumor histology, region, and sample size. Clustering was performed using Pearson correlation and average linkage method.
(TIF)

S2 Figure **Pathway enrichment of meta-signature miRNA targets.** The intensity of color represents the FDR-corrected p-value. Clustering was performed using Pearson correlation and average linkage method.
(TIF)

S1 Checklist **PRISMA Checklist.**
(DOC)
