# Supplementary figures and images for: A Meta-Analysis of MicroRNA Expression in Liver Cancer
Source: PLoS One. 2014 Dec 9;9(12):e114533. doi: 10.1371/journal.pone.0114533 (PMC4260848; doi:10.1371/journal.pone.0114533)

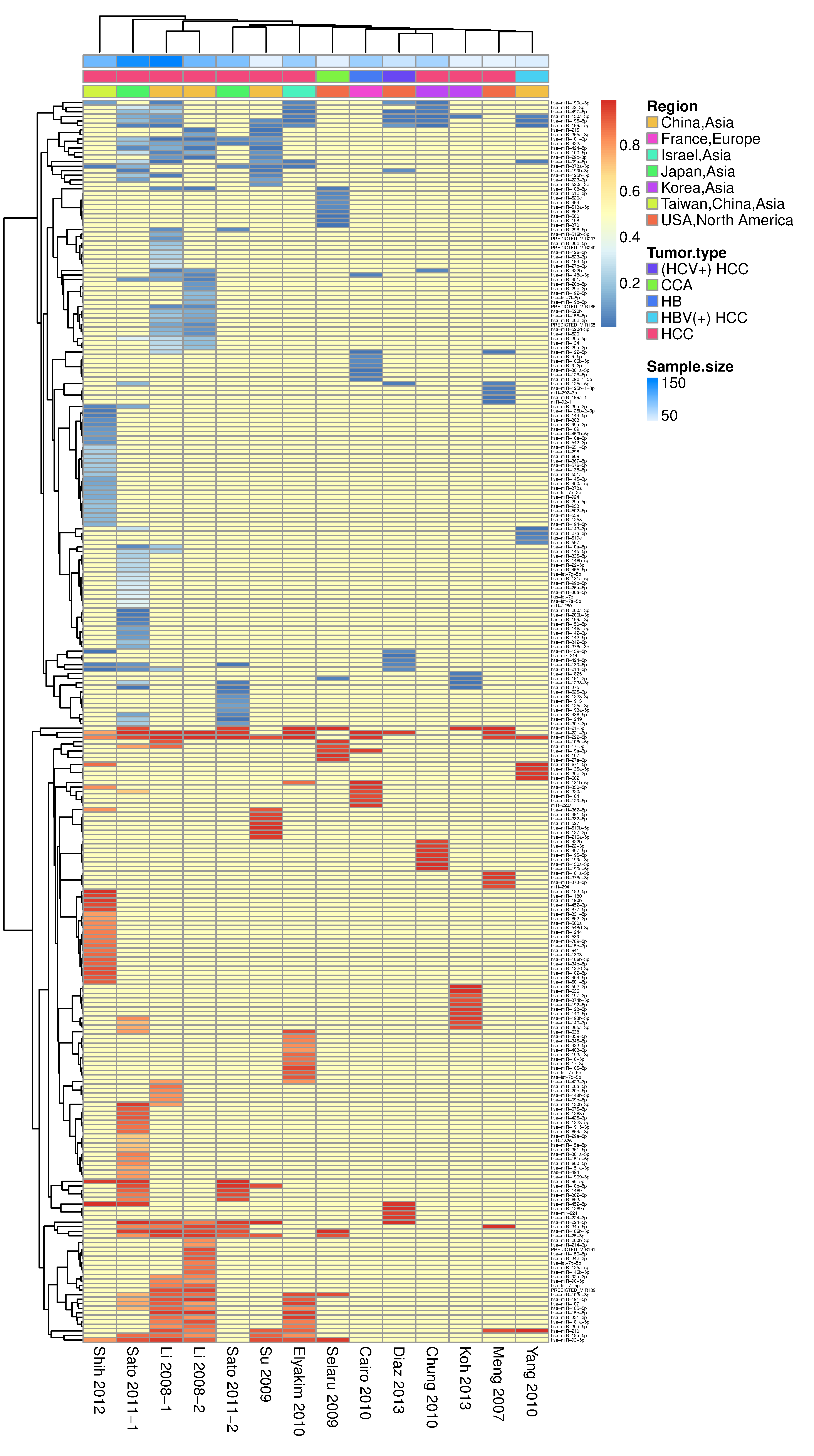

Supplement: S1 Figure — Cluster analysis of miRNA list. Possible correlation was shown according to the subgroups of tumor histology, region, and sample size. Clustering was performed using Pearson correlation and average linkage method. (TIF) [file pone.0114533.s001.tif]

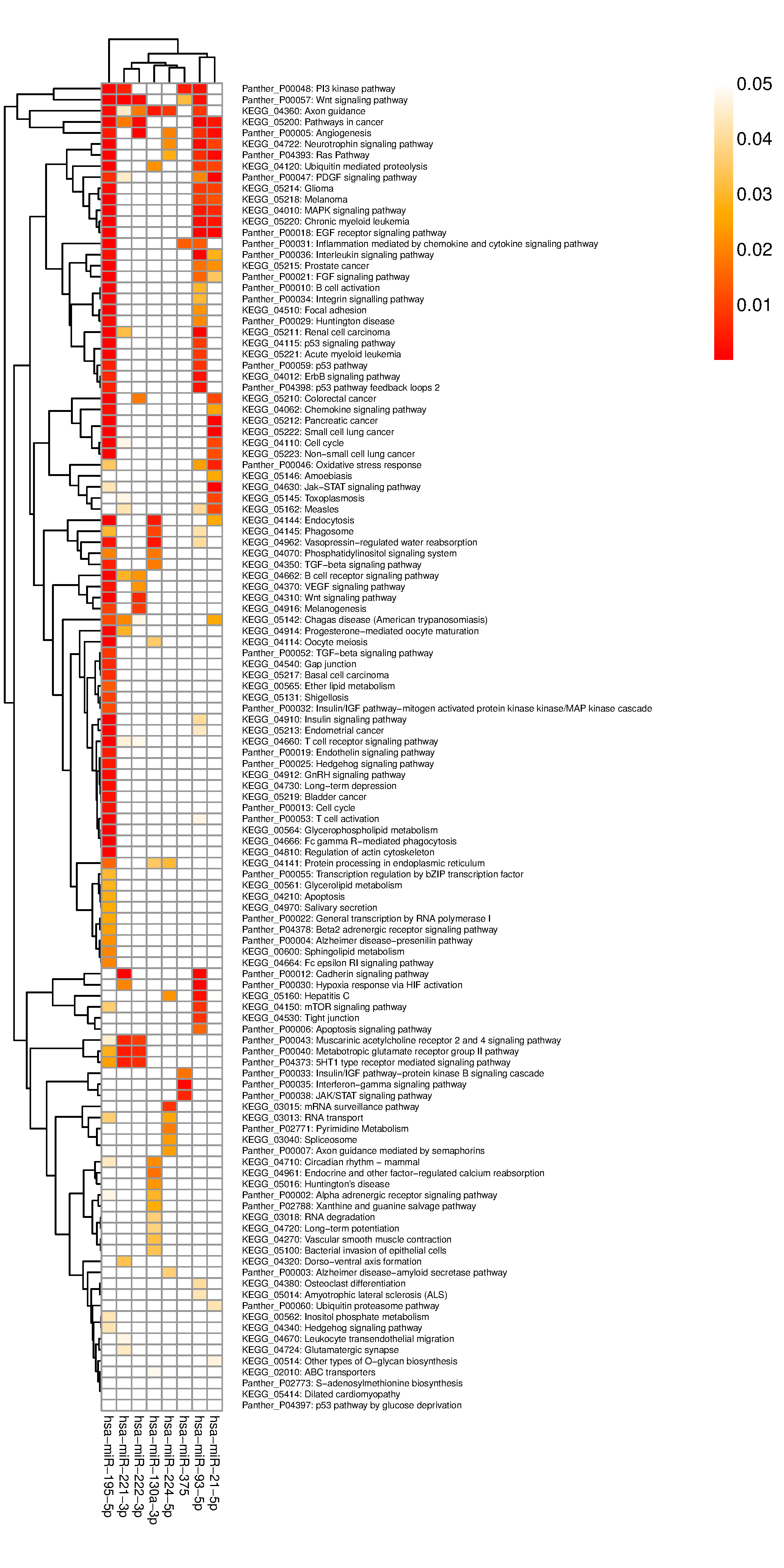

Supplement: S2 Figure — Pathway enrichment of meta-signature miRNA targets. The intensity of color represents the FDR-corrected p-value. Clustering was performed using Pearson correlation and average linkage method. (TIF) [file pone.0114533.s002.tif]
